# Integrating jigsaw teaching into self-regulated learning instruction: an instructional design to improve nursing students’ self-regulated learning

**DOI:** 10.3389/fpubh.2025.1437265

**Published:** 2025-06-11

**Authors:** Yiwen Chen, Jiali Song, Mengying Li, Huijuan He, Xiangrong Wang, Siyu Zhou, Ling Wang

**Affiliations:** ^1^School of Nursing, Hubei University of Chinese Medicine, Wuhan, Hubei, China; ^2^Wuhan Fourth Hospital, Wuhan, Hubei, China; ^3^Department of Nursing, Zhongshan Hospital, Fudan University (Dafeng Branch), Yancheng, Jiangsu, China; ^4^Hubei Shizhen Laboratory, Wuhan, Hubei, China

**Keywords:** jigsaw, self-regulated learning, nursing students, learning engagement, teaching

## Abstract

**Objective:**

This study aimed to investigate the impact of integrating Jigsaw teaching into Self-regulated learning instruction on nursing students’ self-regulated learning ability and engagement.

**Methods:**

This quasi-experimental study was conducted with 94 nursing students from Hubei University of Traditional Chinese Medicine (Hubei, China) in two classes during the fourth education semester. One classroom was assigned to the control group (43) and the other to the intervention group (50). In the course of Basic Nursing, the two classes adopted the traditional lecture teaching method and jigsaw teaching method to teach independent learning, respectively. The entire teaching scheme consisted of 12 class hours. The data collection tools were the self-regulated learning ability scale, the learning engagement scale, Self-assessment and mutual assessment forms. Data was collected before and after the interventions. Finally, data was analyzed by paired *t*-test, independent *t*-test and repeated measurement analysis of variance via SPSS 25.0 software.

**Results:**

The total scores of self-regulated learning ability and learning engagement of nursing students in the Intervention group were better than that of the control group (*p* < 0.05) before and after the educational intervention. The Self-assessment (*F* = 6.778, *p* < 0.001).and group mutual assessment of nursing students (*F* = 11.037, *p* < 0.001) in the Intervention group at three time points would show an increasing trend with prolonging the intervention time.

**Conclusion:**

Jigsaw teaching integrated with self-regulated learning can effectively enhance nursing students’ self-regulated learning ability and commitment, which is worth using in other nursing courses.

## 1 Introduction

Self-regulated learning is the ability of an individual to organize and manage learning activities spontaneously and independently in order to achieve learning objectives and maximize learning effects (1). Individuals with good self-regulate learning abilities can take the initiative to find learning resources, set learning goals, plan time and methods, self-assess and make adjustments timely to their learning strategies (2–4). This competency is particularly critical for nursing professionals, who operate in dynamic healthcare environments requiring continuous knowledge updates and skill refinement to meet evolving patient care standards and clinical demands. For nursing students—future frontline healthcare providers—cultivating robust SRL skills is essential not only for academic success but also for long-term professional competence and adaptability in clinical practice (5, 6).

Self-regulated learning theory believes that learners manage the learning process effectively by actively regulating their thoughts, feelings and behaviors so as to achieve satisfactory learning outcomes and a sense of achievement (7, 8). Based on Self-regulated Learning theory, Zimmerman et al. (9) Proposed a three-stage cyclic model of forethought, performance and self-reflection from the perspective of mutual support of Personality, Behavior and environment factors of social cognitive theory. The model is widely used in education (10–12). Several clinical or classroom teaching studies showed that the self-regulated learning cycle model positively enhanced learners’ self-regulated learning skills (13–16). But now, in traditional classroom teaching, few teaching instructions take full account of environmental factors and are systematically designed using the cyclical effects of personality, behavior and the environment. Although Lan et al. and Baars et al. used the model to support learners’ cognitive (i.e., learning strategies) and metacognitive processes by applying it to informational and networked teaching and learning, using applications to set up learning prompts and learning guidelines that provide a good physical environment and mobilize individual, behavioral factors. However, Lan et al. and Baars et al. applied the model to informational and online teaching. They used apps to set up learning prompts and guidelines to support learners’ cognitive (i.e., learning strategies) and metacognitive processes, providing a good physical environment for learners and mobilizing individual behavioral factors (17–19). However, their cross-disciplinary applicability and scalability remain limited. This gap is especially pronounced in nursing education, where hands-on, group-based learning environments are prevalent, yet underutilized in fostering SRL (20, 21).

Among the environmental factors, apart from physical factors, social factors play an equally important role in the adjustment process of learners’ self-regulated learning. By scientifically designing teaching programs, educators improve social factors’ role in mobilizing students’ individual and behavioral factors. Such teaching programs may be able to use individual, behavioral and environmental factors better to enhance the effectiveness of teaching and learning further. Another study indicated that social factors such as role modeling, peer learning and the support of others help learners to learn by imitation through observing the information, knowledge and experience of others. This facilitates the internalization and translation of learners’ perceptions and behaviors into their own, thus contributing to the development of self-regulated learning. The Jigsaw teaching method, which incorporates role modeling, group work and balanced participation in learning activities, can make full use of social-environmental factors and mobilize the three factors to help each other (22, 23). Meanwhile, the Self-Regulated Learning cycle model can guide learners to set goals, make learning plans, and choose learning strategies in the self-learning process. This model can compensate for the Jigsaw teaching method’s shortcomings in students’ independent learning and self-monitoring.

Despite the recognized importance of SRL in nursing education, few studies have explicitly targeted nursing students or designed interventions tailored to their unique learning needs—such as balancing theoretical knowledge with clinical application and teamwork (24). Therefore, this study integrated jigsaw teaching into the Self-Regulated learning cycle model to form an instructional design for developing Self-regulated learning ability. The research addresses the urgent need for pedagogical strategies that prepare nursing students to thrive in both academic and clinical settings through improved self-regulation and collaborative competence.

## 2 Methods

### 2.1 Research design and setting

This quasi-experimental design was conducted on nursing students from February to March 2023, during the fourth academic semester at Hubei University of Traditional Chinese Medicine (Hubei, China).

### 2.2 Participants

**Figure 1:**
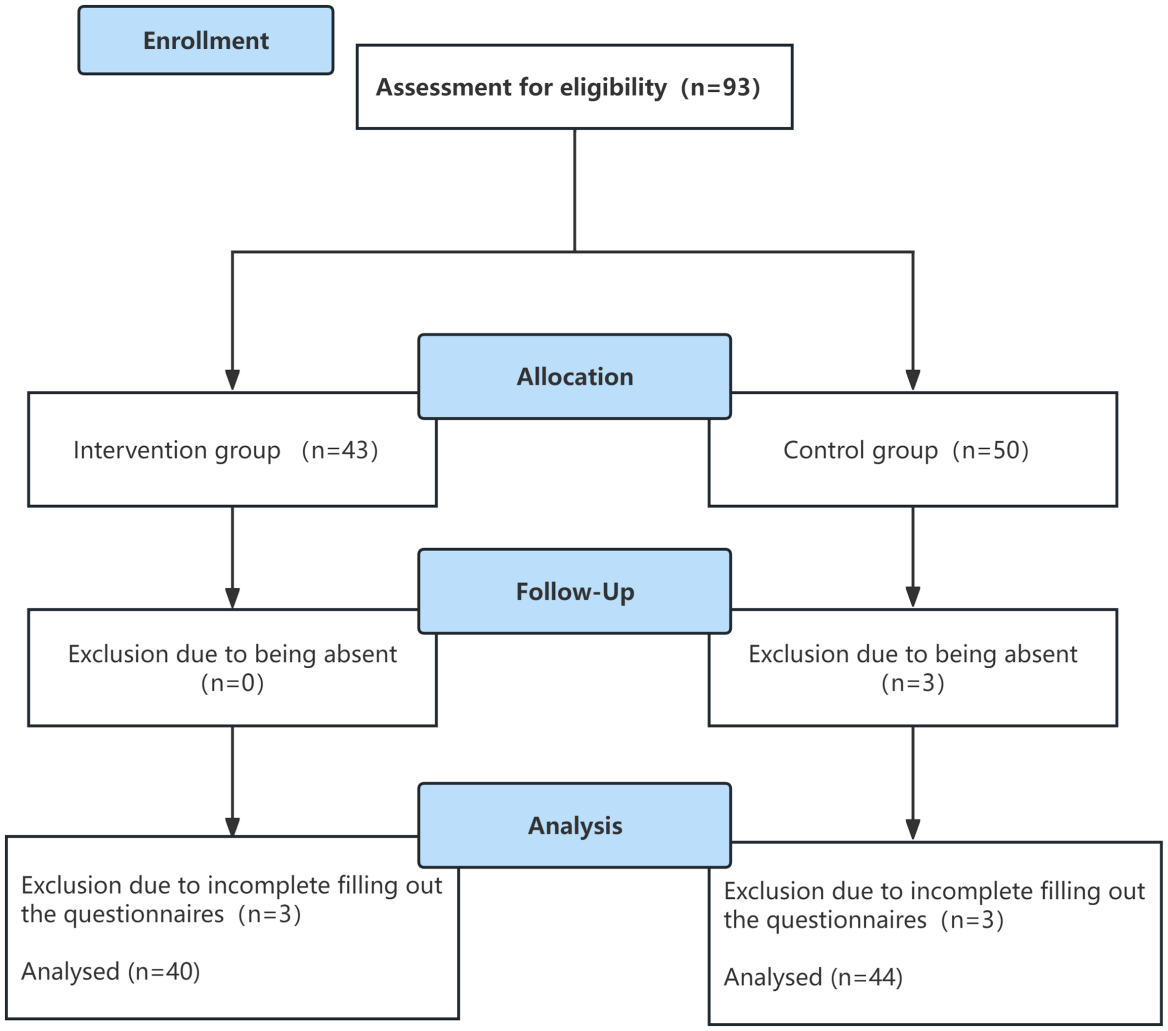
The process of the study.

To calculate the sample size for comparing two sample means, the formula is: n1 = n2 = 2[(Z_α_ + Z_β_)s/*δ*]^2^where the two sample sizes are equal, s represents the estimated standard deviation of the two populations, δ is the difference between the two population means, and Z*_α_* and Z*_β_* are the Z-values corresponding to the significance level α (α = 0.05) and Type II error probability β (β = 0.1), respectively. From the table, Z_α_ = 1.96 and Z_β_ = 1.282. Referring to similar research (25), s was estimated as 6 and *δ* was set at 5.1. Substituting these values into the formula yields n_1_ = n_2_ ≈ 29 for each group. Considering a 20% incidence of dropouts, each group should be recruited to ensure a minimum of 35 participants per group, resulting in a total sample size of at least 70 participants. Participants were recruited from two classrooms of nursing students in the fourth academic semester attending the Basic Nursing course. One classroom was assigned to the control group (43) and the other to the intervention group (50). During the study, three students failed to participate for some reason, and six students were unable to complete the questionnaire. Finally, 84 nursing students entered the control group and the intervention group (Figure 1).

**Inclusion criteria**: ① Nursing students in the fourth academic semester attended the Basic Nursing course at Hubei University of Traditional Chinese Medicine; ② voluntary participation in this study.

**Exclusion criteria**: ① Nursing students with a history of mental illness diagnosis before or during the intervention; ② those absent from the course more than once. ③ Those who filled out the questionnaire incompletely or did the answers regularly.

### 2.3 Process

#### 2.3.1 Teaching and learning arrangements

This study selected three teaching components (care of urination, common blood transfusion reactions and care, penicillin allergy test and management of allergic reactions) suitable for breaking down into multiple sub-tasks from the Basic Nursing course offered in the fourth semester. A total of 12 class hours (6 h online and 6 h offline) were devoted to this study. In this study, teachers in the intervention and control groups were randomly assigned, and the same teaching materials and syllabi were used in both groups. The teachers in both groups were consistent regarding teaching age, educational experience, and the results of the previous teaching evaluation. The teaching activities of the two groups were carried out concurrently. While the control group used traditional teaching methods, the intervention group implemented an instructional design to improve nursing students’ Self-regulated Learning.

#### 2.3.2 Control group

Traditional lecture strategies with interspersed questions and informational interactions were taught to the control group.

#### 2.3.3 Intervention group

**Figure 2:**
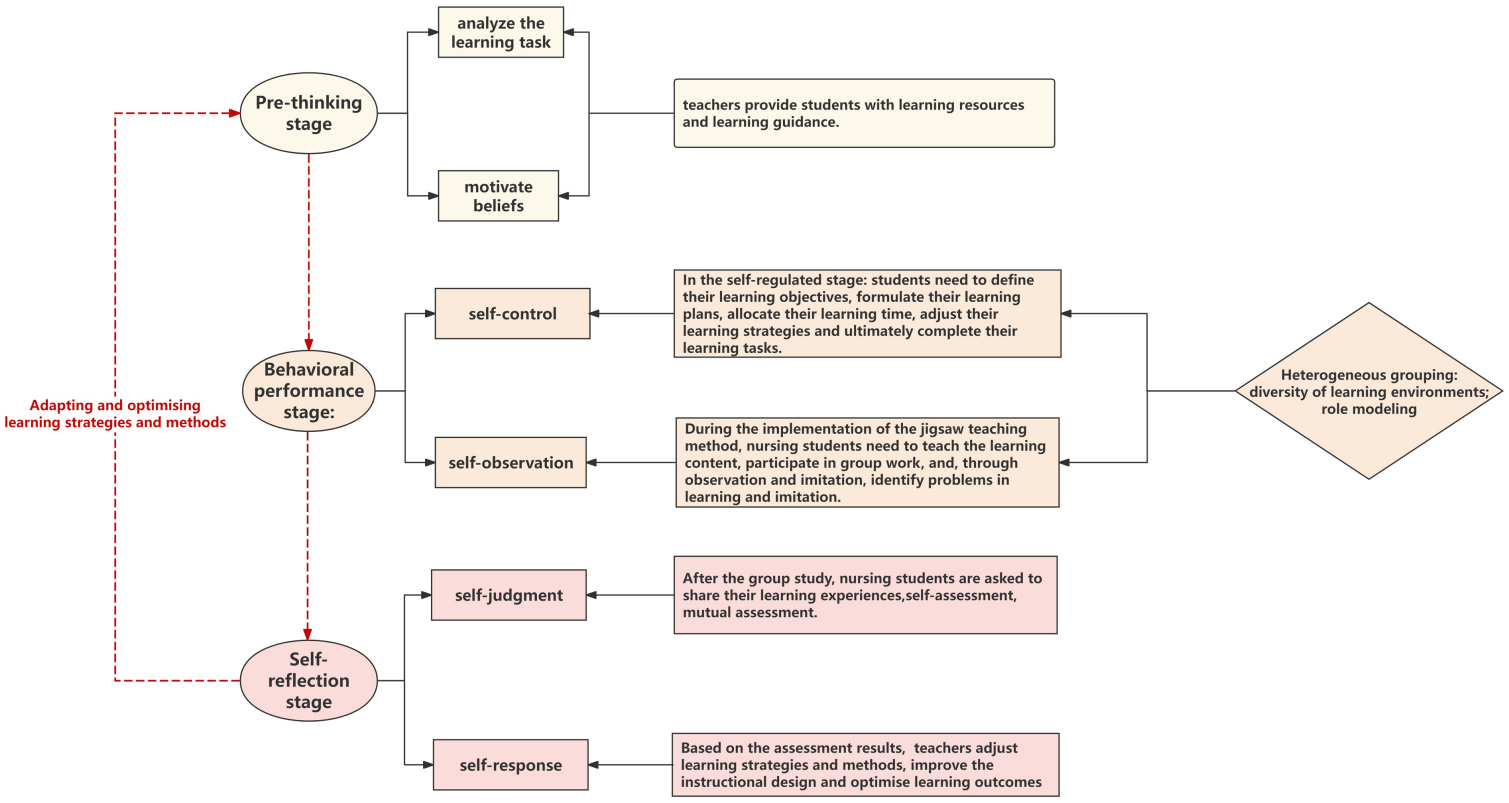
The instructional design for the integration of jigsaw teaching into independent learning.

The intervention group implemented an instructional design to improve nursing students’ Self-regulated Learning. Three content areas were selected for three cycles of instruction. Each cycle consisted of three implementation phases: forethought, performance, and self-reflection. The detailed implementation process is shown in Figure 2.

**Pre-thinking (beforehand) stage**: The purpose of this stage was to help nursing students **analyze the learning task** and **motivate their beliefs**. Self-study stage: Teachers set learning tasks for students and ask them to study the teaching content themselves. At the same time, teachers provided students with learning resources (teaching videos, teaching PPTs) and learning guidance. The learning guide had two parts, including the learning task explanation and learning guidance. The learning task explanation part explained the learning task, teaching process and role setting to students. In the study guide section, teachers guided students on study skills and strategies from the five dimensions of Engage, Explore, Explain, Expand, Elaborate, and Evaluate. These guides helped nursing students clarify learning objectives, develop learning plans, and provide directions and methods for finding teaching resources and resources.

**Behavioral performance (in-progress) stage**: The purpose of this stage was to guide nursing students to self-control and self-observation. In the self-regulated stage, students chose appropriate learning methods and techniques according to their learning objectives and study plans to gain an in-depth understanding and mastery of what they had learned. The Jigsaw method divided students into ‘expert groups’ and ‘jigsaw groups’. ‘In the “expert group” discussion stage, members of the group exchanged and shared what they had learned, understood their learning effect, filled in the gaps in knowledge, identified problems and corrected them in time to ensure the effectiveness of the teaching and learning process. In the ‘jigsaw group’ teaching stage, nursing students took turns teaching other members of the ‘jigsaw group’ the content boards they had learned and tested the learning effect. At the end of the ‘jigsaw group’ teaching, the teacher asked the nursing students who achieved good results to share their learning experiences and strategies.

**Self-reflection (afterward) stage**: The purpose of this stage was to create conditions for self-response and self-judgment for the nursing students. The jigsaw teaching method was completed by organizing the nursing students to share their learning experiences, self-assessment, and mutual assessment. Students make an objective and comprehensive assessment of their learning process, learning strategies and learning outcomes. This assessment could identify their learning strengths and weaknesses and provide guidance and direction for adjustment for the next round of independent learning.

In addition, the teacher progressively increased the task’s difficulty over the 3 cycles of this teaching. Teachers changed from guiding the nursing students to providing counseling and ultimately achieving independent learning, thus promoting the development of nursing students’ independent learning. In addition, the jigsaw method was implemented with the heterogeneous grouping strategy of ‘jigsaw groups’. The heterogeneous grouping of nursing students was based on their third-semester grade point average (divided into four levels: 90–100 points, 80–89 points, 70–79 points, and 69 points and below), gender, and place of origin (in or out of the province).

### 2.4 Instruments

General demographic information included academic performance, place of origin, gender and arts and science division of the study population.

Zhang Xi Yan et al. developed the Self-regulated Learning Ability Scale in 2009 (47). It was mainly used to measure the self-regulated learning ability of nursing students. The scale covered four domains: learning motivation (8 items), self-management ability (11 items), cooperative ability (5 items), and information quality (6 items), with a total of 28 items. The scale was scored with a 5-point Likert scale ranging from total disagreement (score 1) to total agreement (score 5). The total score is 30 to 150. The higher the score of the nursing students, the better their independent learning skills. Cronbach’s *α* coefficient of the scale was 0.82. The Cronbach’s α coefficient for this study was 0.908.

The Learning Engagement Scale was developed by Schaufeli et al. in 2002 (26). This scale assessed students’ psychological states of enduring, positive, and fulfilling emotions and perceptions of learning and research. The scale contains 17 items in three domains: vitality, dedication and concentration. The scoring was done on a Likert 7-point scale ranging from 1 (totally disagree) to 7 (totally agree). The minimum and maximum scores of the scale were reported as 17 to 85, respectively. Cronbach’s *α* coefficient of the Learning Engagement Scale was 0.937. The Cronbach’s α coefficient was 0.955 in this study.

Within the theoretical frameworks of self-regulated learning (SRL) and sociocultural constructivism, the researcher designed the self-assessment form and the mutual assessment form based on a review of the literature (27). The self-assessment form was used to help students find their strengths and room for improvement by reflecting on their performance in group learning. It mainly examines 4 aspects of nursing students’ learning awareness, learning ability, effectiveness, and group cooperation. The mutual assessment form was designed to enhance the effectiveness and quality of whole group learning through evaluation and analysis by enabling students to look at problems from different perspectives and dimensions and to improve teamwork spirit. The mutual assessment form evaluates the rest of the group members in 4 aspects: learning motivation, task completion, teamwork, and expression, and the form calculated the average score of each student. Both were scored on a 100-point scale. Higher scores represent better individual performance or better performance in team learning.

### 2.5 Data collection and analysis

This study employed descriptive and inferential statistical analyses. Pre-study data normality was assessed using the Kolmogorov–Smirnov test, with a significance level set at *p <* 0.05. The mean±standard deviation was used to describe self-regulated learning ability, learning engagement, self-appraisal, and peer evaluation. For between-subjects comparisons, an independent-samples *t-*test was conducted to analyze differences in self-regulated learning ability and learning engagement between the intervention and control groups. Within-subjects changes were evaluated using paired-samples *t*-tests. The self-appraisal and peer evaluation results were analyzed using repeated-measures ANOVA to examine trends in scoring across time points. To control for the family-wise error rate (FWER) resulting from multiple comparisons, the Bonferroni correction was applied. For pairwise comparisons across three time points, the adjusted significance level was set at *α* = 0.0167 (0.05/3). If the assumption of sphericity in repeated measures ANOVA was violated, the Greenhouse–Geisser correction was utilized.

### 2.6 Ethical principles

Participants had the right not to participate or to withdraw from this study at any time and were informed of the study’s purpose, content, scope, and confidentiality. In this study, if a student chooses to withdraw, that student may participate in the traditional teaching method with the control group and will not have their data included in this study. No students in this study refused to participate in the jigsaw method of instruction.

## 3 Results

### 3.1 General information

In the intervention group, there were 40 students, 2 boys (5%) and 38 girls (95%); 16 (40%) in science and 24 (60%) in arts; 25 (62.5%) in the province and 15 (37.5%) outside the province; the average grade in the third semester was (77.98 ± 5.73). In the control group, there were 44 students, 8 (18.2%) males and 36 (81.8%) females; 11 (25%) in science and 33 (75%) in arts; 28 (63.6%) in the province and 16 (36.4%) outside the province; and the average grade in the third semester was (80.16 ± 5.46). The differences between the two groups regarding gender, arts and sciences, place of origin and average grade in the 3rd semester were not statistically significant (*P>*0.05).

### 3.2 Self-regulated learning ability

As shown in Table 1. The independent samples t-test results showed that the difference between the self-regulated ability of nursing students in the intervention and control groups before the intervention was not statistically significant (*p >* 0.05). After the intervention, the difference between the two groups in nursing students’ self-regulated ability was statistically significant (*p <* 0.05). Learning cooperative ability and information literacy were significantly higher than the control group’s (*p <* 0.05). In addition, the paired t-test showed that the scores of nursing students’ self-regulated ability in the intervention group were significantly higher after the intervention than before the intervention (*p <* 0.05), especially in the dimensions of motivation and cooperative ability (*p <* 0.05).

**Table 1:** Comparison of the differences between the intervention and control groups before and after the instructional design of self-regulated learning ability.

Sports event	Control group	Intervention group	*t*	*p*	Cohen’s d
Motivation					
Before intervention	35.43 ± 6.42	34.45 ± 4.85	0.785	0.438	0.171
After intervention	34.20 ± 6.51	36.38 ± 3.66	−1.905	0.061	0.406
*t*	0.853	−3.071			
*p*	0.399	0.004*			
Cohen’s d	0.128	0.486			
Self-management					
Before intervention	31.98 ± 5.47	31.40 ± 4.64	0.519	0.605	0.111
After intervention	30.84 ± 3.92	31.80 ± 3.52	−1.176	0.243	0.257
*t*	1.422	−0.934			
*p*	0.162	0.356			
Cohen’s d	0.119	0.147			
Cooperative ability					
Before intervention	17.14 ± 2.58	17.03 ± 2.44	0.202	0.840	0.043
After intervention	16.70 ± 2.43	17.90 ± 1.65	−2.616	0.011*	0.506
*t*	0.782	−2.617			
*p*	0.438	0.012*			
Cohen’s d	0.118	0.413			
Information quality					
Before intervention	20.75 ± 3.40	20.83 ± 3.06	−0.106	0.916	0.027
After intervention	20.32 ± 3.04	21.80 ± 2.30	−2.532	0.013*	0.546
*t*	0.587	−1.576			
*p*	0.560	0.123			
Cohen’s d	0.088	0.249			
Self-regulated learning ability					
Before intervention	105.30 ± 16.73	103.70 ± 13.58	0.477	0.635	0.104
After intervention	102.07 ± 13.45	107.88 ± 7.67	−2.457	0.017*	0.524
*t*	1.014	−2.712			
*p*	0.316	0.010*			
Cohen’s d	0.153	0.428			

*p < 0.05.

### 3.3 Learning engagement

According to the independent t-test results, the difference between the intervention and control groups of nursing students’ learning engagement and their scores in each dimension before teaching was insignificant (*p* > 0.05). However, after the intervention, the total learning engagement and dedication dimension scores of nursing students in the intervention group were significantly higher than those of the traditional teaching method (*p* < 0.05). Meanwhile, the paired t-test results showed that the intervention of nursing students was better in vitality, dedication and total score after teaching than before teaching, and the differences were statistically significant (all *p* < 0.05; Table 2).

**Table 2:** Comparison of differences between the intervention and control groups before and after the learning engagement intervention.

Sports event	Control group	Intervention group	*t*	*p*	Cohen’s d
Vitality					
Before intervention	25.45 ± 4.17	26.48 ± 5.53	−0.960	0.340	0.209
After intervention	26.89 ± 4.20	28.28 ± 3.58	−1.623	0.109	0.354
*t*	−1.551	−3.257			
*p*	0.128	0.002*			
Cohen’s d	0.233	0.515			
Dedication					
Before intervention	22.48 ± 3.42	23.78 ± 4.53	−1.490	0.140	0.324
After intervention	23.30 ± 3.55	25.20 ± 3.16	0.820	0.011*	0.565
*t*	−1.098	−2.794			
*p*	0.278	0.008*			
Cohen’s d	0.165	0.442			
Concentration					
Before intervention	26.34 ± 4.25	27.60 ± 5.06	−1.239	0.219	0.271
After intervention	26.89 ± 4.44	28.05 ± 2.73	−1.428	0.157	0.312
*t*	−0.578	−0.589			
*p*	0.567	0.560			
Cohen’s d	0.087	0.093			
Learning engagement					
Before intervention	74.27 ± 11.39	77.85 ± 14.38	0.218	0.208	0.277
After intervention	77.07 ± 10.83	81.53 ± 7.35	−2.185	0.032*	0.477
*t*	−1.149	−2.368			
*p*	0.257	0.023*			
Cohen’s d	0.173	0.374			

*p < 0.05.

### 3.4 Self-assessment and mutual assessment of nursing students in the intervention group

Nursing students’ self-assessment and mutual assessment forms were analyzed using repeated measures ANOVA with time as a within-group factor. Post-hoc paired t-tests were conducted to compare scores between specific time points. The results showed a statistically significant difference (*p* < 0.05) between the total self-assessment scale scores and the nursing students’ learning ability and group cooperation dimensions at the three time points. It indicated that the nursing students’ self-assessment would tend to increase with prolonging the intervention time (*p <* 0.05). The differences between the total scores of the mutual assessment scale and the dimensions of learning motivation and task completion of the nursing students at the three time points were also statistically significant (*p <* 0.05; Table 3). This suggested a gradual increase in the total scores of the mutual assessment form and the scores of the dimensions of motivation to learn and task completion of the nursing students with time.

**Table 3:** Comparison of scores on self-assessment form and mutual assessment form of nursing students on 3 occasions of jigsaw methods.

Sports event	1st-Excretion	2nd-Blood transfusion	3rd-Penicillin	F	*p*
Self-assessment					
Awareness of learning	14.95 ± 1.80	15.65 ± 2.18	15.78 ± 2.34	2.150	0.123
Learning ability	14.13 ± 2.09	15.25 ± 2.15①	15.75 ± 3.13①	5.709	0.005
Learning effect	14.70 ± 2.70	15.35 ± 2.87	15.70 ± 2.41	1.780	0.175
Group work*	30.87 ± 4.30	31.68 ± 4.62	32.37 ± 4.25①	3.969	0.027
Total score*	74.65 ± 7.73	77.93 ± 9.35①	79.60 ± 8.27①②	6.778	0.000
Mutual assessment					
Learning motivation*	17.40 ± 0.75	18.34 ± 0.87①	18.54 ± 0.99①	15.809	0.000
Completion of the mandate	17.31 ± 0.81	17.84 ± 1.09①	18.02 ± 0.95①	7.721	0.001
Teamwork*	35.61 ± 3.35	35.72 ± 2.21	36.05 ± 3.29	1.523	0.231
Power of expression	17.43 ± 1.18	17.48 ± 0.81	17.81 ± 0.84①②	2.847	0.064
Totals	87.75 ± 4.04	89.38 ± 2.92①	90.42 ± 4.26①	11.037	0.000

① *p < 0.05* compared with the 1st lecture; ② *p < 0.05* compared with the 2nd lecture; *represents that the spherical hypothesis was not satisfied.

## 4 Discussion

### 4.1 The instructional design of jigsaw teaching to develop self-regulated learning ability helped to strengthen nursing students’ self-regulated learning ability

Self-regulated learning ability plays an important role in effectively promoting personal growth and professional development of nursing professionals and is an important support for realizing lifelong learning (28). The comparative results of this study found that the intervention group of nursing students’ motivation, ability to cooperate in learning and total scores were significantly higher than those before teaching. After the intervention, the dimensions of learning cooperative ability and total scores of nursing students in the intervention group were significantly higher than those of the control group. The results indicated that incorporating the jigsaw method into the SRL (Self-regulated Learning) cycle model significantly affected nursing students’ self-directed learning skills. Compared with SANAIE et al. (29), who used 14 h to implement Jigsaw teaching methods only, the implementation of Jigsaw teaching methods under the guidance of the SRL cycle model was able to enhance the nursing student’s self-directed learning ability rapidly in a short period. This is closely related to the ability of the SRL cycle model to combine SRL theory and social cognitive theory to provide a structured learning path for nursing students (14, 30).

The SRL cycle model emphasizes the importance of the interplay of personal, behavioral and environmental factors in enhancing learners’ self-regulated learning (7, 8, 23). The instructional design based on the SRL cyclic model provided nursing students with learning guidance in the pre-reflection phase. It enabled them to clarify learning objectives, formulate learning plans, develop learning strategies, and motivate learning in the individual dimension. At the behavioral performance stage, nursing students retrieve learning materials on the online platform according to the learning objectives, analyze and evaluate the information obtained, and organize and summarize what they have learned. Nursing students built their knowledge framework to enhance their information literacy level further. The jigsaw teaching method is a unique group-supportive learning method. The jigsaw teaching method can provide nursing students with a social environment with role modeling, teaching support and feedback. This enables nursing students to continuously optimize their learning performance through observation, evaluation, and imitation (31, 32). This instructional design set up a self-reflection stage, where the teacher organized self-assessment, mutual assessment, and learning experience sharing for the nursing students after each implementation of the jigsaw teaching method. In this process, nursing students could receive positive feedback to enhance their motivation and self-efficacy. Nursing students could also evaluate and learn the results, continuously adjust and improve their learning strategies, optimize the learning process, and further enhance their independent learning ability (33, 34). Although the difference in the dimensions of self-management ability was insignificant in this study, it could be seen from the data that the post-intervention data was higher than the pre-intervention data. As self-management was an ongoing process, extending the intervention time to a later stage was recommended to check the intervention’s effect.

In summary, integrating the jigsaw teaching method into the SRL cycle model could not only fill the gap of the jigsaw teaching method in the pre-teaching stage of the inability to provide learning guidance for nursing students. It could also use the unique teaching advantages of the jigsaw teaching method, give full play to the role of social and environmental factors, mobilize the role of the three factors of mutual assistance, and rapidly improve the independent learning ability of nursing students.

### 4.2 The instructional design of jigsaw teaching to develop self-regulated learning ability helped to increase nursing students’ learning engagement

The results of this study showed that the intervention group of nursing students scored significantly higher in dedication, vigor dimension, and total learning engagement after teaching than before teaching. The dedication dimension and total learning engagement scores were better than the control groups. In the jigsaw teaching method, each nursing student was required to undertake the assigned task alone. This form of division of labor and cooperation easily stimulated the nursing students’ sense of responsibility, so they would participate in learning more actively and positively, complete their own tasks and contribute to the group. It was consistent with the findings of Toyokawa et al. (35) and Qin et al. (36). Cooperative learning and mutual support in the jigsaw teaching method could promote cooperation and emotional communication among students, establish a good learning atmosphere, develop a sense of responsibility and participation, and mobilize a reasonable balance between collaboration and competition. It could have a positive impact on students’ learning engagement (29, 37). Guided by the SRL cycle model, this instructional design added heterogeneous grouping and instructional strategies such as experience sharing, self-assessment, and mutual assessment. This could give students exemplary role models and diverse learning experiences to enhance social influence (38). At the same time, it could also provide nursing students with timely explanation, guidance and feedback, stimulate healthy competition among peers, improve the accuracy of imitation behaviors, and then obtain positive feedback, which positively affected learning vitality and dedication (23, 39, 40). In this instructional design, the teacher provided learning guidance for students, which could help the nursing students generate learning path deviation in self-regulated learning to reach the learning objectives quickly and effectively (41, 42). It could be seen that combining the jigsaw teaching method with the SRL cycle model so that the advantages of both complement each other positively enhanced the learning engagement of nursing students. However, due to the short duration of the intervention and the absence of relevant intervention content, there was no obvious advantage in improving the concentration of nursing students. In later studies, appropriate interventions can be developed to enhance the concentration of nursing students.

### 4.3 The instructional design of jigsaw teaching to develop self-regulated learning ability helped to improve the general learning skills of nursing students

The results of this study showed that the experimental group’s total self-assessment and mutual-assessment scores at the three time points tended to increase continuously, especially in the areas of learning ability and group cooperation in the self-assessment and learning motivation and task completion in the mutual assessment. This indicated that the instructional design of jigsaw teaching integrated with self-regulated learning teaching could effectively enhance nursing students’ learning motivation and task completion. The instructional design of this study not only provided nursing students with a structured learning path and a framework for self-regulated learning but also provided them with a diverse form of metacognitive supervision. Such a self-learning structure could help nursing students recognize and monitor their learning process, improve their ability to use learning strategies and facilitate the integration and construction of knowledge, thus enhancing learning outcomes (43, 44). However, the teamwork and presentation skills differences were not statistically significant in the group mutual assessment. However, the presentation of the data showed that the trend was still increasing. This indicated that the group learning approach of verbally expressing one’s opinions and thoughts was beneficial in improving nursing students’ expressive and communication skills (45, 46). In addition, the heterogeneous grouping strategy was useful for nursing students to develop a cooperative spirit and a sense of teamwork that supports and promotes each other at different stages of learning.

## 5 Limitations

There were some limitations to this study. One, this study was implemented for only three rounds (a total of 12 class hours), which limited the improvement of the self-management skills of the nursing students, and it was recommended that the intervention be extended at a later stage. Secondly, as this study was conducted based on the normal teaching program of the school, the study participants were also involved in other courses and activities during the same period. Therefore, the influence of confounding factors on the findings could not be completely ruled out in this study. Thus, an independent instructional intervention program could be explored later by increasing the time and hours of instructional delivery for a more in-depth study.

## 6 Conclusion

The instructional design of jigsaw teaching integrated with self-regulated learning instruction could fully exploit social factors so that the three personal, behavioral, and environmental factors can interact in the physical classroom. Furthermore, nursing students’ self-regulated learning ability, learning engagement, and comprehensive learning ability could be rapidly improved. Therefore, the results of this study provide a useful reference for nursing professional education and can provide educators with effective teaching methods and strategies.

## Data Availability

The original contributions presented in the study are included in the article/supplementary material, further inquiries can be directed to the corresponding author.
